# Identification of the molecular mechanism and diagnostic biomarkers in the thoracic ossification of the ligamentum flavum using metabolomics and transcriptomics

**DOI:** 10.1186/s12860-020-00280-3

**Published:** 2020-05-13

**Authors:** Jiahao Li, Lingjia Yu, Shigong Guo, Yu Zhao

**Affiliations:** 1 Department of Orthopaedic Surgery, Peking Union Medical College Hospital, Peking Union Medical College and Chinese Academy of Medical Science, No. 1 Shuaifuyuan Dongdan, Dongcheng District, 100730 Beijing, P.R. China; 2 National Spinal Injuries Centre, Stoke Mandeville Hospital, Aylesbury, UK

**Keywords:** Ossification of the ligamentum flavum, Uric acid, XDH, Metabolomics, Transcriptomic

## Abstract

**Background:**

To establish a metabolite fingerprint of ossification of the thoracic ligamentum flavum (OTLF) patients using liquid chromatography-mass spectrometry (LC-MS) in combination with transcriptomic data and explore the potential molecular mechanism of pathogenesis.

**Results:**

The study cohort was composed of 25 patients with OTLF and 23 healthy volunteers as a control group. Thirty-seven metabolites were identified out by UPLC-MS including uric acid and hypoxanthine. Nine metabolites, including uric acid and hypoxanthine, were found with a Variable Importance in Projection (VIP) score over 1 (*p* < 0.05). Pathway enrichment indicated that purine metabolism pathways and the other four metabolism pathways were enriched. Transcriptomic data revealed that purine metabolism have a substantial change in gene expression of OTLF and that xanthine dehydrogenase (XDH) is the key regulatory factor. Receiver operating characteristic (ROC) analysis indicated that 17 metabolites, including uric acid, were found with an AUC value of over 0.7.

**Conclusion:**

Uric acid might be the potential biomarker for OTLF and play an important role within the detailed pathway. XDH could affect purine metabolism by suppressing the expression of hypoxanthine and xanthine leading to low serum levels of uric acid in OTLF, which could be a focal point in developing new therapeutic methods for OTLF.

## Background

Ossification of the ligamentum flavum (OLF) primarily occurs in the thoracolumbar spine, especially at T9-T11, and almost all OLF cases were exclusively reported in East Asian countries [1, 2]. The overall occurance rate in China is 3.8% of the general population in 2010 and it is more common in females [3]. The first case of OLF was reported by Polgar in 1920 and was recognized as a cause of myeloradiculopathy [3]. OLF is a relatively rare disease and numerous studies have focused on its progression at both histopathological and cellular levels. OLF is often accompanied by other spinal degenerative diseases [4], which means the recognition of its clinical manifestation, diagnosis and progression is extremely difficult. Imaging studies such as magnetic resonance imaging (MRI) and computed tomography (CT) are the most commonly employed methods for radiological diagnosis [5]. As for the treatment of OTLF, surgical treatments are the first choice for patients due to the progressive nature of the disease and poor outcomes from conservative management [6]. The most common surgical strategy is posterior decompression as it is the most effective [7], however this still has its limitations in that patients with multilevel OTLF cannot have the same outcome as those with single- or dual-level lesions [2].

Metabolomics also knowns as metabolic profiling is used in the detection of smaller molecules with a molecular weight of less than 1800 Da [8]. Metabolomics can be used to investigate disease or systemic health from bio-samples such as body fluid and tissue [9]. It is an effective approach to detect both intra- and extra-cellular processes through analyzing the metabolites which are produced by various biochemical pathways [10]. Nontargeted and targeted analyses are two main approaches in metabolomics detection. Targeted analysis is focused on a specific compound set such as amino acids [11], lipids [12] and energy metabolism [13]. In contrast, untargeted approaches aim to find potential biomarkers for the diagnosis of diseases and therefore it needs to measure thousands of molecules at a time [14]. In order to analyze the metabolites in samples, separation techniques such as chromatography (both liquid and gas) and capillary electrophoresis are needed and these techniques are often combined with mass spectrometry to confirm the molecular weight, or used in nuclear magnetic resonance (NMR) to confirm specific molecular structures.

The studies of the diagnosis and treatment methods of OTLF mostly focus on the radiological features. There have been some other studies exploring the pathogenesis of OTLF such as mechanical [15], degenerative [16] and genetic factors [17] but detailed clinical progression of the disease continues to be poorly understood. Although there have been several studies that have analyzed biomarkers for ossification of the spinal ligament (OSL) and found calcium-phosphate metabolism markers, bone turnover markers, sclerostin and so on, there are no definitive conclusions to date [18]. Few studies focus on the metabolism dysfunction in OTLF, one previous research indicated that lectin metabolism might affect the progress of OTLF [19] and thus further research on metabolism disorder may be needed to develop earlier diagnosis and new therapeutic methods.

In our current study, UPLC-MS system based untargeted metabolomic approaches were applied to find potential biomarkers in the OTLF serum samples, and transcriptomic techniques were also ultilized to identify and study the regulator gene associated with the metabolic pathways found in the metabolomic analysis. Findings from these approaches may provide useful information for the diagnosis and treatment of OTLF.

## Results

### Demographic and clinical features

The study cohort was composed of 25 patients with OTLF and 23 healthy volunteers as a control group. The mean age of OTLF patients was 53.88 ± 9.46 years and the mean age of the control group was 53.71 ± 9.77 years indicating no significant difference. Specific patient demographic and clinical features are illustrated in Table 1.

**Table 1:** Demographic and clinical features

Patient No.	Gender	Age	Diagnosis
T1	M	35	T11-T12
T2	M	36	T7-10
T3	M	38	T2-T7
T4	M	40	T3-T5,T10-T11
T5	M	43	T9-L1
T6	M	47	T7-T11
T7	F	50	T9-T11
T8	M	50	T5-T7
T9	M	52	T4-T9
T10	F	52	T9-T11
T11	F	53	T3-T10
T12	F	54	T9-T11
T13	F	58	T2-T3
T14	F	58	T9-L1
T15	F	59	T5-L1
T16	M	59	T8-L5
T17	F	59	T1-T2
T18	F	60	T4-T5
T19	M	60	T2-T3
T20	M	61	T1-T3,T9-T12
T21	F	62	C4-T4
T22	M	65	T3-T4
T23	F	65	T3-T5
T24	F	65	T10-L1
T25	F	66	T9-T11
C1	M	59	–
C2	M	35	–
C3	M	40	–
C4	M	50	–
C5	M	59	–
C6	M	37	–
C7	F	50	–
C8	F	50	–
C9	F	41	–
C10	F	60	–
C11	M	62	–
C12	F	62	–
C13	M	45	–
C14	M	63	–
C15	F	65	–
C16	F	63	–
C17	F	37	–
C18	F	58	–
C19	M	64	–
C20	M	59	–
C21	F	51	–
C22	M	55	–
C23	F	61	–

*T* thoracic ossification of ligamentum flavum, *C* control

### Metabolite identification

**Fig. 1:**
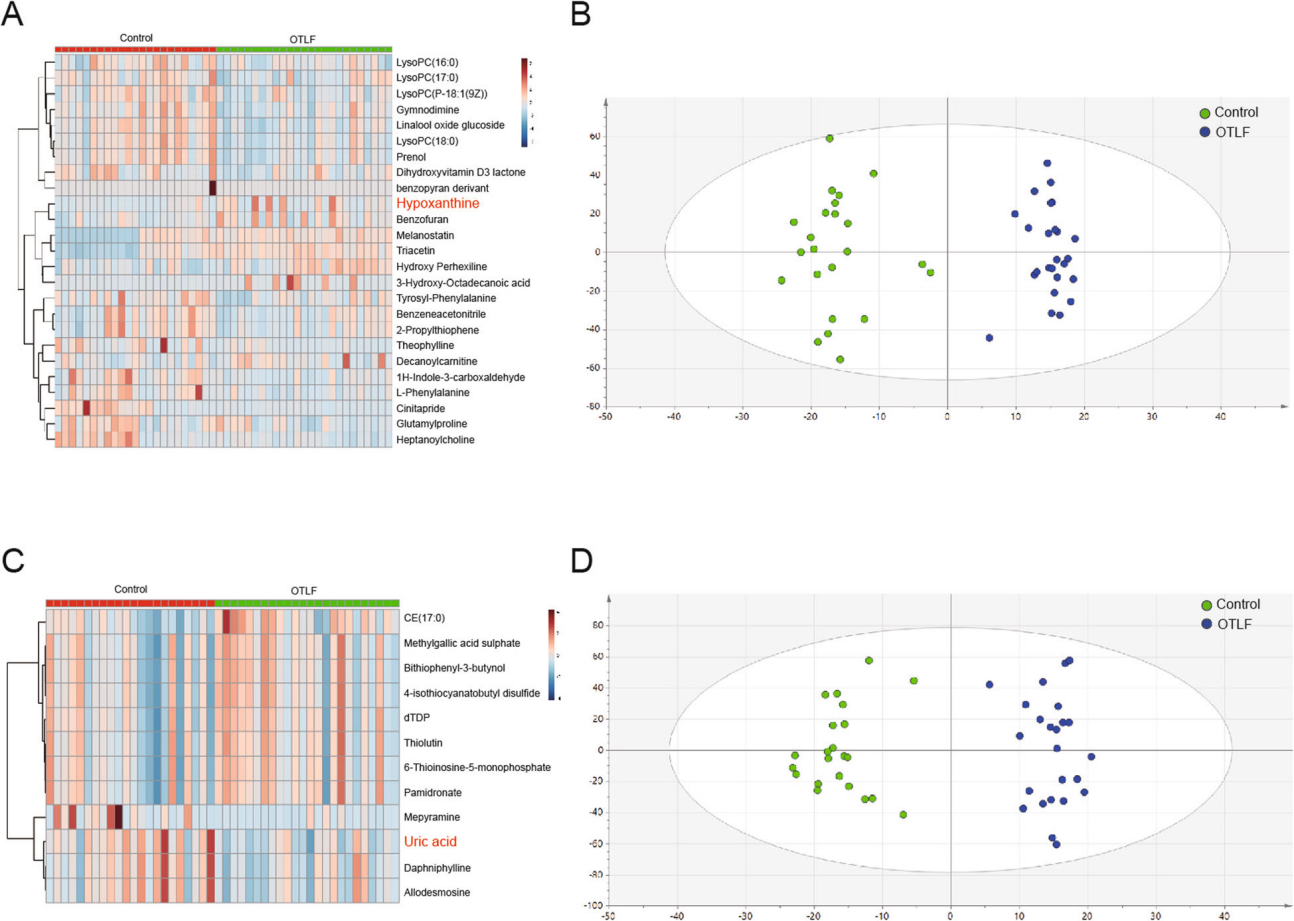
**a**: The heatmap of metabolites detected by positive ion mode. **b**: The OPLS-DA model of positive ion mode; green dots represent the control group and blue dots represent the OTLF group. **c**: The heatmap of metabolites detected by negative ion mode. **d**: The OPLS-DA model of negative ion mode; green dots represent the control group and blue dots represent the OTLF group

**Fig. 2:**
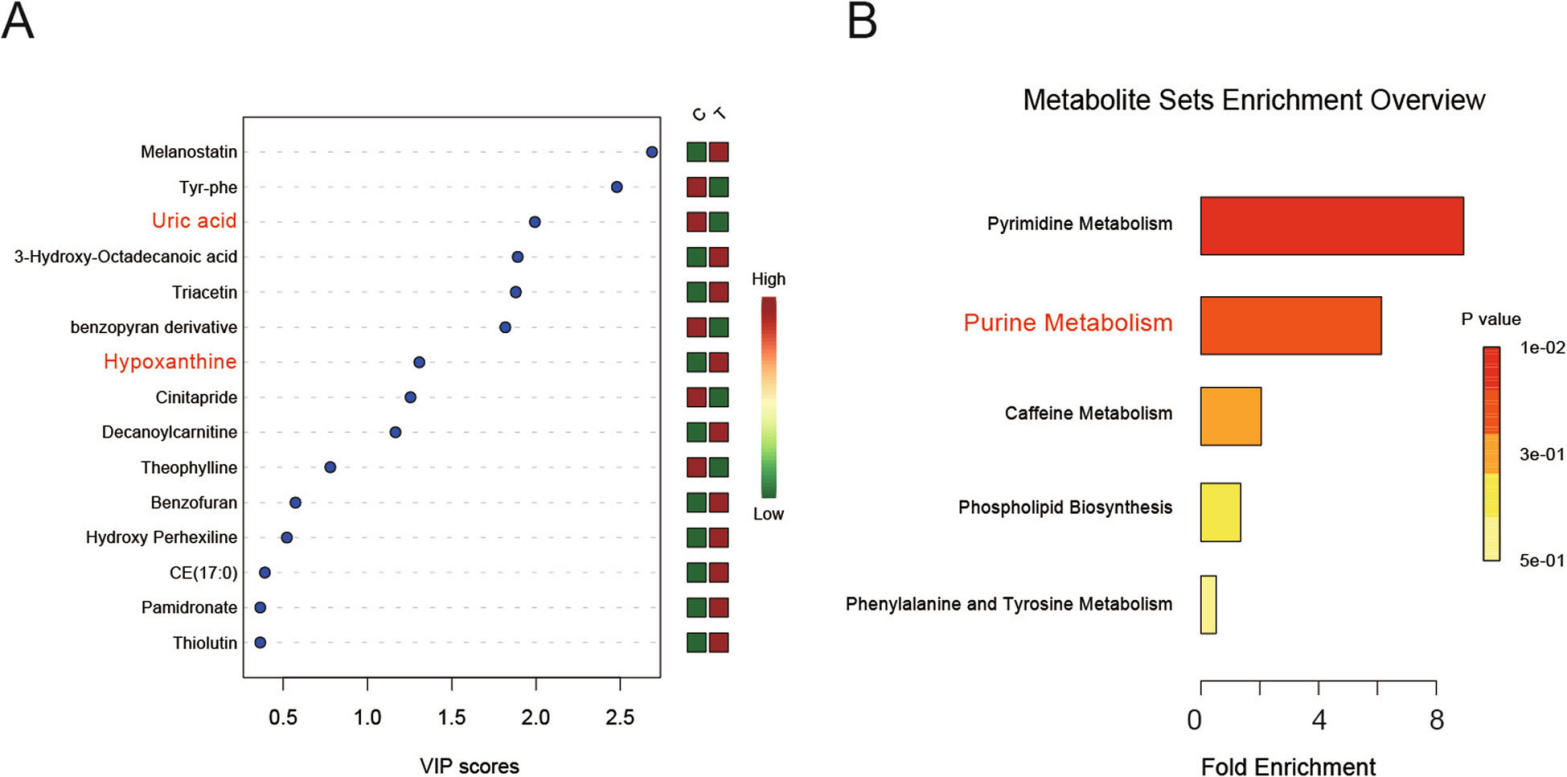
**a**: The VIP scores of metabolites, 9 metabolites with the scores over 1.0 which suggestive a high impact on the process of OTLF. **b**: Metabolite sets enrichment analysis which is based on the KEGG pathway

A total of 37 metabolites were found in the samples of the two groups. Twenty-five metabolites were detected under the positive mode mass spectrometry (Fig. 1a) and 12 metabolites were found under the negative mode (Fig. 1c). PCA and OPLS-DA analysis were used to identify the metabolite and its phenotype. As shown in the PCA score plots (Fig. 1b&d), the serum samples within each group were closely clustered into each other in both positive and negative ion mode, while samples from different groups were clearly separated. Nine metabolites (including uric acid, triacetin and hypoxanthine) were found to have a Variable Importance in Projection (VIP) score over 1 (*p* < 0.05) (Fig. 2a). In order to comprehend the relation between differential metabolites and the metabolic pathways, a metabolite sets enrichment analysis was carried out, which showed out of the five metabolic pathways that underewent enrichment analysis, the pyrimidine and purine metabolism pathways showed significant differences in OTLF (Fig. 2b).

### Transcriptomic data

**Fig. 3:**
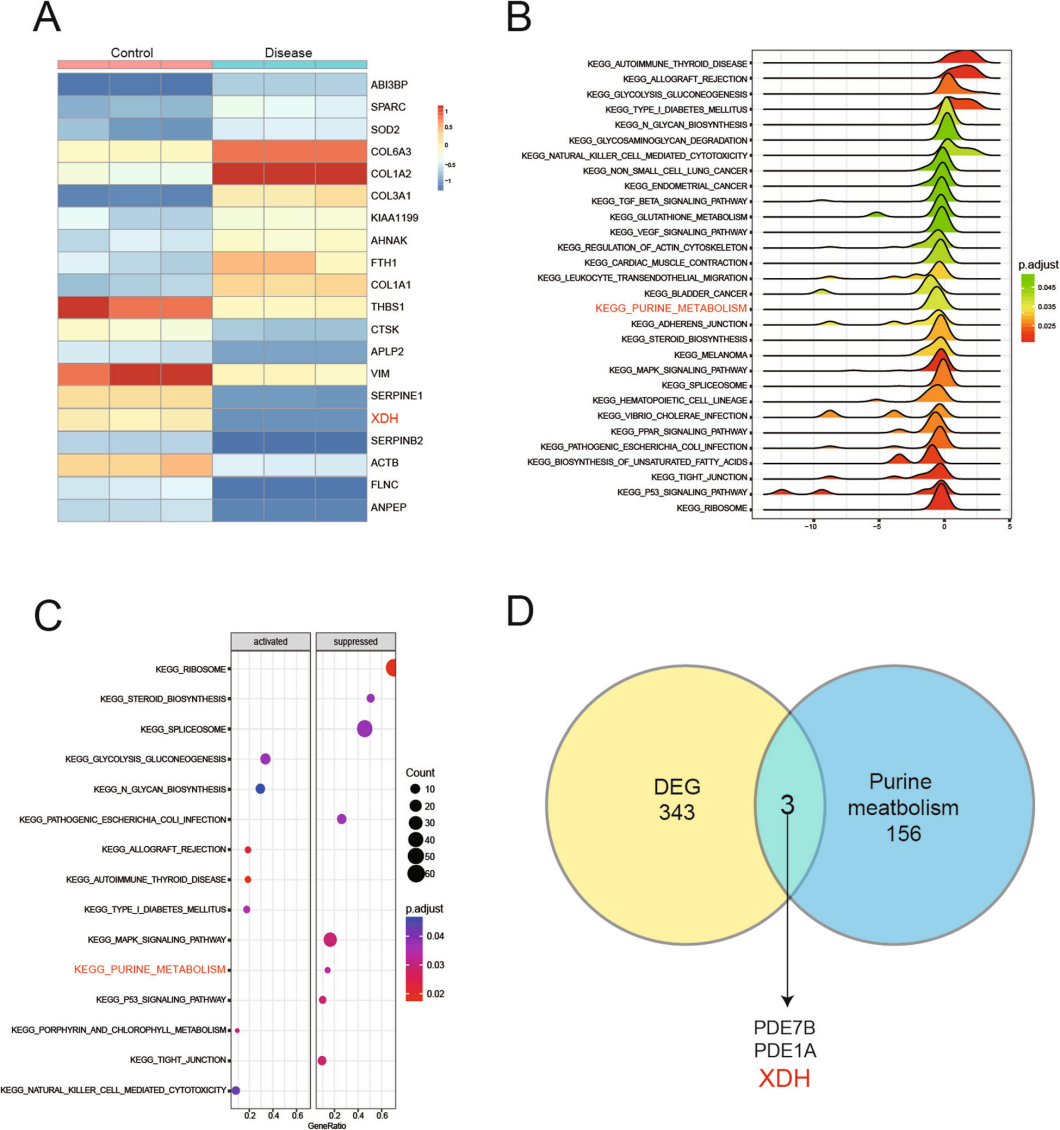
**a**: The heatmap of the differentially expressed genes. The top 10 up-and down-regulated genes were listed on the heatmap. **b**: Joyplot of the 7 up-regulated metabolism pathway and 23 down-regulated pathways. **c**: Dotplot referred to the same results of joyplot and the enrichment degree. **d**: Venn diagram indicating the common gene between extremely differential expression genes and all genes involved in purine metabolism. It showed that 3 genes were the common genes

Transcriptomic data GSE69787 was acquired from GEO platform and it was used to perform that the relationship between genes and the metabolites in process of ligament ossification. Three thousand fifty-six genes were found have notable differences between two groups (student t’test *p* < 0.05, FDR < 0.05). Heatmap illustrated the top ten up- and down-regulated genes in two groups (Fig. 3a) and these genes were enriched to the metabolism pathway by Gene Set Enrichment Analysis (GSEA). Density joyplot and dotplot were plotted according to the results of GSEA and it showed 30 pathways had significant impact on the process of ligament ossification (Fig. 3b) and 8 metabolic pathways were suppressed in patients while 7 were activated (Fig. 3c). Combined with metabolite results, purine metabolism was also found have a substantial change in gene expression. 346 differential expression genes were selected under the condition of *p* < 0.0001, FDR < 0.05 to make the intersection with genes on the purine metabolism pathway. As shown in the Venn diagram in Fig. 3d common genes were found which were PDE7B, PDE1A and XDH .

### Potential biomarker and related pathway analysis

**Fig. 4:**
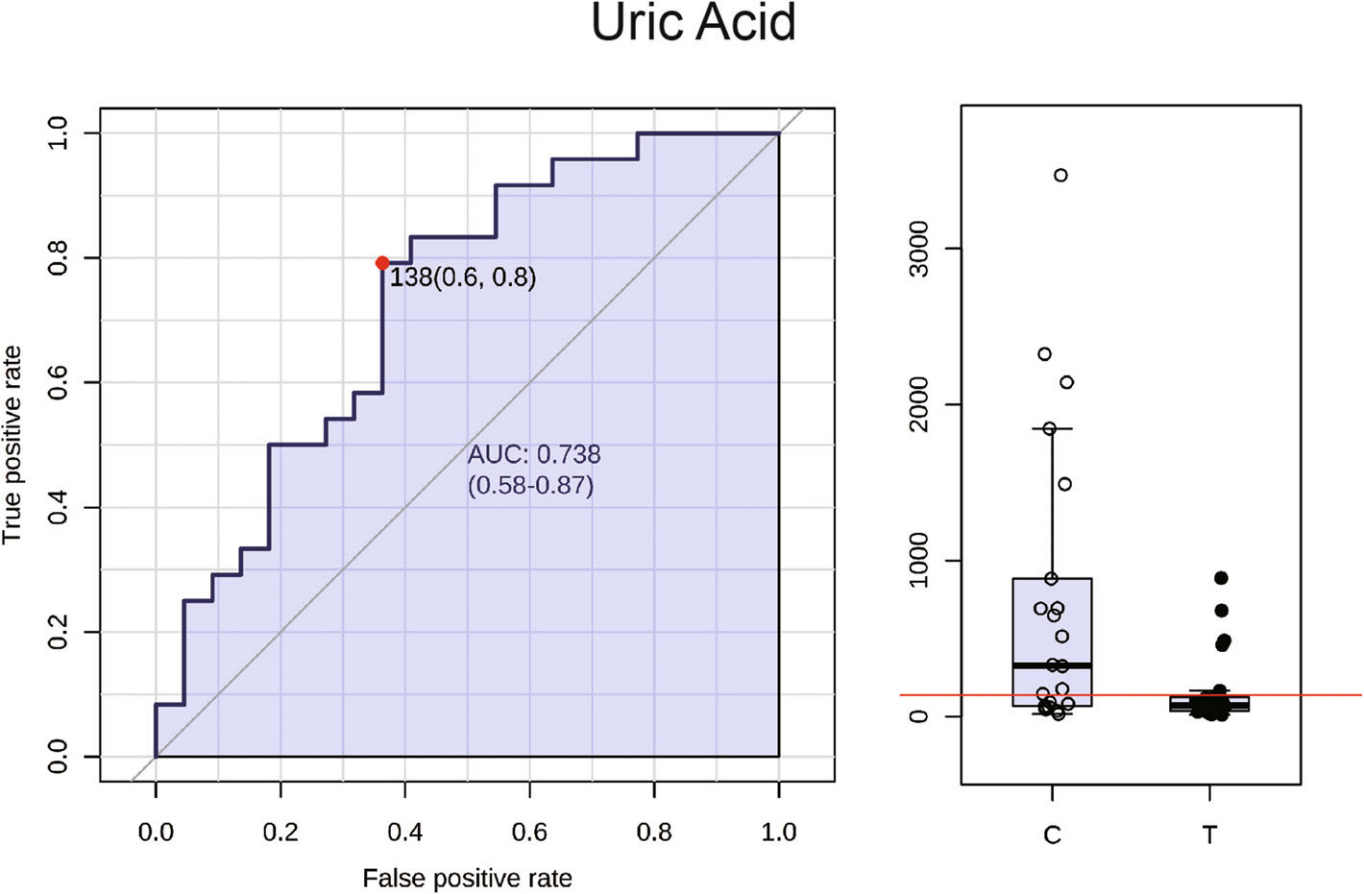
The ROC curve of uric acid and the AUC value was 0.738. The boxplot showed significant decrease in patient group

**Fig. 5:**
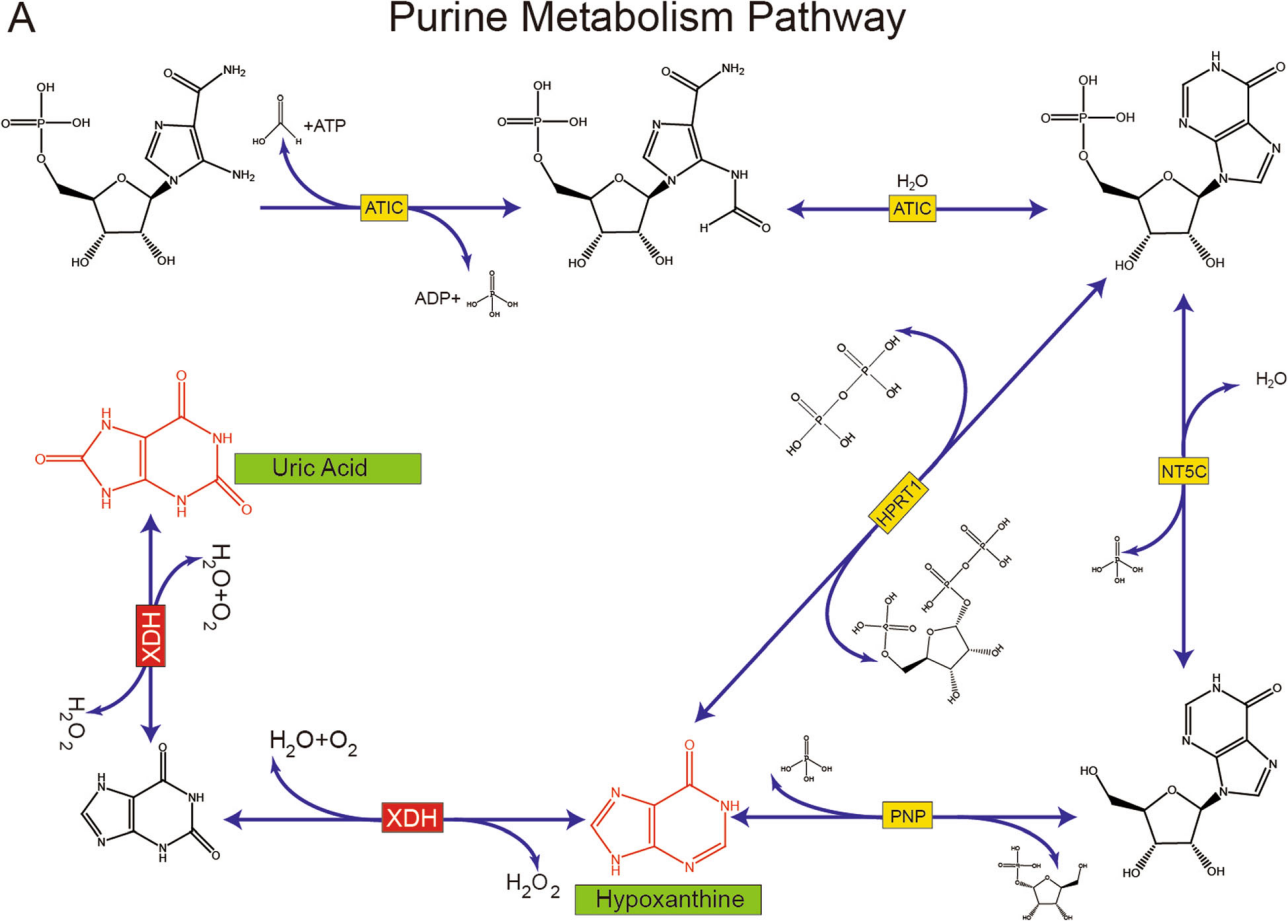
A part of purine metabolism pathway. The yellow box indicated the regulated gene on this part of reaction. The differentially expressed metabolites were shown in red. The related differentially expressed gene was showed in the red box

Receiver operating characteristic curves (ROC) were plotted to inspect the Area Under Curve (AUC) which is an effective and combined measure of sensitivity and specificity that describes the inherent validity of diagnostic tests. A total 17 metabolites were found with an AUC value (Table 2) of over 0.7 which included uric acid (Fig. 4) suggesting that the uric acid may be the potential biomarker for OTLF. Figure 5 illustrates that the relationship between XDH, uric acid and hypoxanthine within the purine metabolism pathway. The metabolism of hypoxanthine was regulated by the gene XDH and the concentration of uric acid was also regulated by XDH.

**Table 2:** Metabolites profile

Metabolites	logFC	P.Value	FDR	AUC
Triacetin	1.153904	1.33E-05	0.000164	0.827381
Mepyramine	−0.13584	0.042187	0.086717	0.815476
Prenol	−0.17288	0.140915	0.217244	0.803571
Cinitapride	−1.24341	2.54E-06	9.39E-05	0.801587
cis_Hydroxy Perhexiline	0.013212	0.778289	0.832752	0.789683
L_Phenylalanine	−0.20394	0.005179	0.031935	0.781746
LysoPC(18:0)	−0.12575	0.028497	0.080608	0.779762
Daphniphylline	−0.11042	0.010347	0.045228	0.771825
Allodesmosine	−0.04424	0.131739	0.217244	0.769841
Heptanoylcholine	−0.0631	0.52506	0.626685	0.765873
LysoPC(P_18:1(9Z))	−0.07243	0.012224	0.045228	0.761905
CE(17:0)	0.130081	0.016951	0.057018	0.751984
1H_Indole_3_carboxaldehyde	−0.17782	0.037036	0.080608	0.728175
Linalool oxide glucoside	−0.01237	0.817001	0.839695	0.720238
Uric acid	−1.18616	0.034894	0.040608	0.718254
Gymnodimine	−0.13483	0.036976	0.080608	0.710317
Melanostatin	2.068298	8.87E-06	0.000164	0.700397
Benzeneacetonitrile	−0.06204	0.338149	0.446839	0.696429
Dihydroxyvitamin D3 lactone	−0.24723	0.136295	0.217244	0.696429
2_Propylthiophene	−0.04659	0.650347	0.751963	0.694444
3_Hydroxy_Octadecanoic acid	1.114997	0.030578	0.080608	0.684524
Pamidronate	0.026685	0.164965	0.234757	0.680556
Tyrosyl_Phenylalanine	−0.97294	0.034981	0.080608	0.680556
Bithiophenyl_3_butynol	0.019384	0.183398	0.251324	0.678571
Thiolutin	0.128482	0.002532	0.01876	0.678571
6_Thioinosine_5_monophosphate	0.002499	0.787738	0.832752	0.676587
Benzofuran	0.267858	0.002535	0.01876	0.674603
dTDP	−0.00273	0.92585	0.92585	0.674603
Hypoxanthine	0.850452	0.010926	0.045228	0.670635
LysoPC(17:0)	−0.1121	0.371349	0.468671	0.670635
Glutamylproline	0.046695	0.380004	0.468671	0.668651
4_isothiocyanatobutyl disulfide	0.002576	0.718373	0.805449	0.662698
benzopyran derivant	−0.76542	0.15302	0.22647	0.65873
Methylgallic acid_O_sulphate	0.046586	0.113276	0.202466	0.65873
Decanoylcarnitine	0.719299	0.011702	0.045228	0.638889
LysoPC(16:0)	−0.10209	0.064155	0.124932	0.638889
Theophylline	−0.59669	0.114913	0.202466	0.623016

*FDR* false discovery rate, *AUC* Area Under Curve

## Discussion

OLF and calcification of the ligamentum flavum (CLF) are differential diagnoses in patients with posterior extradural compressive lesions. They have similar clinical features, but different pathological manifestations. OLF tends to arise from the lateral capsular portion of the ligamentum flavum and is continuous with the bony laminae [20]. Hypertrophic and degenerative changes of elastic fiber are accompanied by ossified lesions predominantly at the surface of the capsular portion. However, CLF is a crystal deposition disease that mainly affects the central portion of the ligamentum flavum. Calcium deposits mainly occur in the central part of the ligamentum flavum, which is surrounded by degenerated elastic fibers [21]. CT can facilitate the detection of characteristic findings and help distinguish between CLF and OLF [21]. In our study, all patients were diagnosed with thoracic OLF rather than CLF.

OLF and ossification of the posterior longitudinal ligament (OPLL) are diseases which occur ossification in spinal ligament. Both of them result in the compression of the spinal cord and nerve roots. Thus patients may have a similar clinical history that a slowly progressive of neurological symptoms from discomfort to severe myelopathy. And Kawaguchi et al. suggested that over half of the patients with cervical OPLL had OLF [22]. However, there are some differences between the two diseases. The most frequent level of OPLL was at C5 vertebral level while OLF was predominant at upper and lower thoracic levels [23]. In our study, only 2 of 25 patients combined with OPLL. Thus our conclusion only applies to OTLF. Although some studies reported the potential pathogenesis of OTLF such as mechanical, degenerative and genetic factors, the causes of the disease continues to be poorly understood. As far as we know, this study is the first to discover XDH might participate in the progress of OTLF.

In our study, we identified 37 metabolites in the serum of all participants based on the UPLC-MS platform by using untargeted approaches. Both groups demonstrated clusters separated in OPLS-DA models. Metabolite enrichment analysis indicated that the purine metabolism pathway has changed greatly in OTLF patients. ROC analysis indicated that uric acid with the AUC value of over 0.7 might have a diagnosis value in OTLF. Combined with transcriptomic data, 3056 different expression genes were detected and GSEA indicated that purine metabolism might have an impact on the process of OTLF. KEGG pathway suggested that low expression of XDH might increase the level of hypoxanthine and suppress the hypoxanthine metabolism to uric acid that leads to the low level of uric acid in the OTLF patients. All these revealed that uric acid might be the potential biomarker for OTLF.

Previous studies report that osteosis has a relation with metabolism. Davis et al. have suggested that heterotopic ossification might be affected by several metabolism pathways such as tricarboxylic acid (TCA) cycle, amino acid metabolism and lipolysis [24]. Ma et al. suggested that amino acid metabolism and lipid metabolism plays an important role in bone resorption and bone formation through the regulation by strontium salt [25]. Both studies indicate that metabolomics techniques can be used to explore the pathogenesis of OTLF. Using metabolomics related techniques, Sohn et al. suggested that bone mineral density has a positive correlation with OTLF [26]. Fan et al. concluded that obesity was a risk factor for OTLF after determining that leptin-stimulated cell osteogenesis is regulated by STAT3, Runx2 and steroid receptor coactivator-1 [19]. Several studies have focused on the element metabolites related to bone remodeling, but to the best of our knowledge, there have been no previous studies exploring serum metabolites in OTLF. We have found that the downregulation of gene XDH might be the possible mechanism of OTLF, might provide a novel direction of treatment. XDH catalyzes the successive oxidation of hypoxanthine to xanthine and xanthine to uric acid [27]. The decrease of XDH expression leads to the accumulation of hypoxanthine and the decrease of uric acid. Uric acid has been studied extensively in other fields such as Parkinson disease [28], cardiovascular function [29]. It has also been studied in bone formation, by stimulating osteoblasts and osteoclasts. Yan et al. suggested that acid was negatively correlated with bone formation markers in postmenopausal females [30]. One possible explanation could be that the low level of uric acid might promote bone formation, especially the heterotopic ossification. Future studies could explore the relationship between uric acid and heterotopic ossification.

Our study’s main limitation is the relatively small sample size, and thus further studies with a larger sample size could be considered, especially focusing on the metabolites of the ligament tissue and targeted metabolic approaches. Despite this limitation, our study of OTLF based on metabolomics is the first study of its kind and offers a new diagnostic approach in the early stage of OTLF.

## Conclusion

To the best of our knowledge, this is the first study of the relationship of the metabolome with OTLF. We have found that the process of OTLF may be associated with the level of hypoxanthine and uric acid. Uric acid might be the potential biomarker for OTLF and play an important role within the detailed pathway. XDH may regulate the level of uric acid through the purine metabolism pathway and this new discovery may in turn provide a new direction for the diagnosis and treatment of OTLF.

## Methods

### Sample selection

Serum samples were obtained from an experimental group of 25 patients with OTLF and a control group of 23 healthy volunteers. All samples were acquired from Peking Union Medical College Hospital. Written informed consent was obtained from all patients and the study was approved by the hospital institutional review board (JS-981). The inclusion criterion of the experimental group was a confirmed radiological diagnosis of OTLF. All patients had a slowly progressive history of neurological symptoms and signs which is suspected to be caused by thoracic stenosis (TSS). The axial plain CT scan of the thoracic vertebrae and sagittal reconstruction provides analysis of the morphology and density of ossification. MRI can show signal changes and deformation of the spinal cord. The combination of these two imaging examination methods can differentially diagnose OTLF from other causes of TSS (such as OPLL). The exclusion criteria include (1) participants had any history of spinal deformity, other spinal disease or trauma were removed; (2) participants had any systemic metabolic diseases (such as skeletal fluorosis) were removed; (3) participants who were not willing to sign the written consent form were removed; (4) the cases were removed where clinical information was lacking or missing and therefore statistical analysis could not be performed. The healthy controls were recruited from among healthy subjects during an annual health check at Peking Union Medical College Hospital. Standard meals was provided 3 days before sampling.

### Sample treatment

Morning fasting blood samples were taken from a peripheral vein and collected into ethylenediaminetetraacetic acid (EDTA) tubes. Samples were stored at 4 °C for 3 h before centrifugating at 3000 r/min at room temperature for 20 min. The supernatants were separated and stored at − 80 °C until further analyses.

Before further analysis, the samples were thawed at room temperature. 100 μL serum was extracted and mixed with 400 μL methanol. The mixture was vortexed for 5 min and then centrifuged at 13000 r/min for 15 min. 400 μL of supernatants were then taken for UPLC-MS analysis.

### UPLC-MS analysis

UPLC-MS analysis was performed using a Waters ACQUITY system (Waters Corporation, Milford, USA) combined with a Thermo Scientific high-resolution mass spectrometer (MS) system (Thermo Fisher Scientific Inc., San Jose, USA). C18 column (Acquity UPLC BEH C18–2.1 × 100 mm, 1.7 μm) was used to achieve the separation purpose. 0.2% formic acid solution was used for mobile phase A, pure acetonitrile was used for mobile phase B. Linear gradient elution methods was used for separation. Each sample was injected three times with 5 μL on each occasion. The temperature of the column oven was held at 40 °C and the flow rate was controlled at 0.4 mL/min. Both positive and negative ion mode was applied for data collection of MS.

### Metabolites and Transcriptomic data analysis

MarkerLynx (Waters, USA) was used for peak finding, filtering and alignment in the original spectrums. SIMCA-P + 12.0 (Umetrics, Umea, Sweden) was used to perform statistical analyses between two groups such as principal component analysis (PCA) and orthogonal partial least squares (OPLS). MedCalc® Version 11.4.2.0 software was used to plot the receiver operating characteristic curve (ROC) analysis. The differential metabolites statistic analyses were carried out by SPSS 16.0. Metabolites were mapped to the KEGG (Kyoto Encyclopedia of Genes and Genomes Database) [31] and HMDB (The Human Metabolome Database) [32]. Transcriptomic data GSE69787 was acquired from GEO (Gene Expression Omnibus) platform [33]. R project [34] was used to ascertain the differentially expressed metabolites and genes. Volcano plot and heatmaps were also carried out through R project.

## Data Availability

The datasets used during the current study are available from the corresponding author on reasonable request.

## Abbreviations

OTLF: Ossification of the Thoracic Ligamentum Flavum
OLF: Ossification of the Ligamentum Flavum
OSL: Ossification of the Spinal Ligament
CLF: Calcification of the Ligamentum Flavum
TSS: Thoracic Stenosis
OPLL: Ossification of the Posterior Longitudinal Ligament
LC-MS: Liquid Chromatography-Mass Spectrometry
UPLC-MS: Ultra-high Performance Liquid Chromatography-Mass Spectrometry
PCA: Principal Component Analysis
OPLS: Orthogonal Partial Least Squares
KEGG: Kyoto Encyclopedia of Genes and Genomes
VIP: Variable Importance in Projection
XDH: Xanthine Dehydrogenase
ROC: Receiver Operating Characteristic
AUC: Area Under Curve
MRI: Magnetic Resonance Imaging
CT: Computed Tomography
NMR: Nuclear Magnetic Resonance

## References

[1] Fong SY, Wong HK (2004). Thoracic myelopathy secondary to ligamentum flavum ossification. Ann Acad Med Singap.

[2] Gao R, Yuan W, Yang L, Shi G, Jia L (2013). Clinical features and surgical outcomes of patients with thoracic myelopathy caused by multilevel ossification of the ligamentum flavum. Spine J.

[3] Guo JJ, Luk KD, Karppinen J, Yang H, Cheung KM (2010). Prevalence, distribution, and morphology of ossification of the ligamentum flavum: a population study of one thousand seven hundred thirty-six magnetic resonance imaging scans. Spine (Phila Pa 1976).

[4] Inamasu J, Guiot BH (2006). A review of factors predictive of surgical outcome for ossification of the ligamentum flavum of the thoracic spine. J Neurosurg Spine.

[5] Zhou SY, Yuan B, Chen XS, Li XB, Zhu W, Jia LS (2017). Imaging grading system for the diagnosis of dural ossification based on 102 segments of TOLF CT bone-window data. Sci Rep.

[6] Yang Z, Xue Y, Zhang C, Dai Q, Zhou H (2013). Surgical treatment of ossification of the ligamentum flavum associated with dural ossification in the thoracic spine. J Clin Neurosci.

[7] Kang KC, Lee CS, Shin SK, Park SJ, Chung CH, Chung SS (2011). Ossification of the ligamentum flavum of the thoracic spine in the Korean population. J Neurosurg Spine.

[8] Monteiro MS, Carvalho M, Bastos ML, Guedes de Pinho P (2013). Metabolomics analysis for biomarker discovery: advances and challenges. Curr Med Chem.

[9] De Cecco CN, Caruso D, Schoepf UJ, Wichmann JL, Ter Louw JR, Perry JD, Picard MM, Schaefer AR, Parker LW, Hardie AD (2016). Optimization of window settings for virtual monoenergetic imaging in dual-energy CT of the liver: a multi-reader evaluation of standard monoenergetic and advanced imaged-based monoenergetic datasets. Eur J Radiol.

[10] Martin FP, Ezri J, Cominetti O, Da Silva L, Kussmann M, Godin JP, Nydegger A. Urinary metabolic phenotyping reveals differences in the metabolic status of healthy and inflammatory bowel disease (IBD) children in relation to growth and disease activity. Int J Mol Sci. 2016;17(8):1310. 10.3390/ijms17081310 PMC5000707 27529220

[11] Jing F, Hu X, Cao Y, Xu M, Wang Y, Jing Y, Hu X, Gao Y, Zhu Z (2018). Discriminating gastric cancer and gastric ulcer using human plasma amino acid metabolic profile. IUBMB Life.

[12] Bhattacharyya S, Pence L, Yan K, Gill P, Luo C, Letzig LG, Simpson PM, Kearns GL, Beger RD, James LP (2016). Targeted metabolomic profiling indicates structure-based perturbations in serum phospholipids in children with acetaminophen overdose. Toxicol Rep.

[13] Zhang X, Lin Q, Chen J, Wei T, Li C, Zhao L, Gao H, Zheng H. High glucose-induced cardiomyocyte death may be linked to unbalanced branched-chain amino acids and energy metabolism. Molecules. 2018;23(4):807. 10.3390/molecules23040807 PMC6017930 29614759

[14] Zhang X, Quinn K, Cruickshank-Quinn C, Reisdorph R, Reisdorph N (2018). The application of ion mobility mass spectrometry to metabolomics. Curr Opin Chem Biol.

[15] Cai HX, Yayama T, Uchida K, Nakajima H, Sugita D, Guerrero AR, Yoshida A, Baba H (2012). Cyclic tensile strain facilitates the ossification of ligamentum flavum through beta-catenin signaling pathway: in vitro analysis. Spine (Phila Pa 1976).

[16] Lang N, Yuan HS, Wang HL, Liao J, Li M, Guo FX, Shi S, Chen ZQ (2013). Epidemiological survey of ossification of the ligamentum flavum in thoracic spine: CT imaging observation of 993 cases. Eur Spine J.

[17] Liu Y, Zhao Y, Chen Y, Shi G, Yuan W (2010). RUNX2 polymorphisms associated with OPLL and OLF in the Han population. Clin Orthop Relat Res.

[18] Kawaguchi Y (2019). Biomarkers of ossification of the spinal ligament. Global Spine J.

[19] Fan D, Chen Z, Chen Y, Shang Y (2007). Mechanistic roles of leptin in osteogenic stimulation in thoracic ligament flavum cells. J Biol Chem.

[20] Ono K, Yonenobu K, Miyamoto S, Okada K (1999). Pathology of ossification of the posterior longitudinal ligament and ligamentum flavum. Clin Orthop Relat Res.

[21] Takahashi T, Hanakita J, Minami M (2018). Pathophysiology of calcification and ossification of the ligamentum flavum in the cervical spine. Neurosurg Clin N Am.

[22] Kawaguchi Y, Nakano M, Yasuda T, Seki S, Hori T, Kimura T (2013). Ossification of the posterior longitudinal ligament in not only the cervical spine, but also other spinal regions: analysis using multidetector computed tomography of the whole spine. Spine (Phila Pa 1976).

[23] Kawaguchi Y, Nakano M, Yasuda T, Seki S, Hori T, Suzuki K, Makino H, Kimura T (2016). Characteristics of ossification of the spinal ligament; incidence of ossification of the ligamentum flavum in patients with cervical ossification of the posterior longitudinal ligament - analysis of the whole spine using multidetector CT. J Orthop Sci.

[24] Davis EL, Salisbury EA, Olmsted-Davis E, Davis AR (2016). Anaplerotic accumulation of tricarboxylic acid cycle intermediates as well as changes in other key metabolites during heterotopic ossification. J Cell Biochem.

[25] Ma B, Li X, Zhang Q, Wu D, Wang G, Jiye A, Sun J, Li J, Liu Y, Wang Y (2013). Metabonomic profiling in studying anti-osteoporosis effects of strontium fructose 1,6-diphosphate on estrogen deficiency-induced osteoporosis in rats by GC/TOF-MS. Eur J Pharmacol.

[26] Sohn S, Yoon JW, Chung CK (2014). Increased bone mineral density in patients with ossification of the ligamentum flavum: a case-control study. J Clin Densitom.

[27] Wang CH, Zhang C, Xing XH (2016). Xanthine dehydrogenase: an old enzyme with new knowledge and prospects. Bioengineered.

[28] Kim IY, O'Reilly EJ, Hughes KC, Gao X, Schwarzschild MA, Hannan MT, Betensky RA, Ascherio A (2018). Integration of risk factors for Parkinson disease in 2 large longitudinal cohorts. Neurology.

[29] Kim SC, Shah NR, Rogers JR, Bibbo CF, Di Carli MF, Solomon DH (2018). Assessment of coronary vascular function with cardiac PET in relation to serum uric acid. PLoS One.

[30] Yan DD, Wang J, Hou XH, Bao YQ, Zhang ZL, Hu C, Jia WP (2018). Association of serum uric acid levels with osteoporosis and bone turnover markers in a Chinese population. Acta Pharmacol Sin.

[31] Kyoto Encyclopedia of Genes and Genomes Database. http://www.genome.jp/kegg/kegg1.html. 2018. Accessed 25 Apr 2018.

[32] The Human Metabolome Database. http://www.hmdb.ca/. 2018.

[33] Gene Expression Omnibus. https://www.ncbi.nlm.nih.gov/geo/. 2018. Accessed 25 Apr 2018.

[34] R project. https://www.r-project.org/. 2018. Accessed 25 Apr 2018.

